# The workers opinions have a value in the Code of Ethics: Analysis of the contributions of workers in virtual Forum Catalan Institute of Health

**DOI:** 10.1186/s12910-015-0081-1

**Published:** 2015-12-23

**Authors:** Eva Peguero, Anna Berenguera, Enriqueta Pujol-Ribera, Begoña Roman, Carmen M. Prieto, Núria Terribas

**Affiliations:** Centre d’Atenció Primària El Castell. Carrer de Guillermo Marconi, 9, 08860 Castelldefels, Àmbit d’Atenció Primària Metropolitana Sud. Institut Català de la Salut, Barcelona, Spain; Unitat de recerca del Institut Universitari d’investigació en Atenció Primària Jordi Gol (IDIAP- Jordi Gol), Gran Via de les Corts Catalanes, 587, àtic, Barcelona, 08007 Spain; Universitat Autònoma de Barcelona, Edifici A. Rectorat, Bellaterra (Cerdanyola del Vallès), 08193 Spain; Gerència d’Àmbit d’Atenció Primària, Balmes, 22, 3ª planta, Barcelona, 08007 Spain; Facultat de Filosofia, Universitat de Barcelona, Edifici B, Campus UAB, Bellaterra, (Cerdanyola del Vallès), 08193 Spain; Centre d’Atenció Primària El Plà, Plaça Felip Alcantara s/n, 08980 Sant Feliu de Llobregat. Àmbit d’Atenció Primària Metropolitana Sud. Institut Català de la Salut, Barcelona, Spain; Institut Borja de Bioètica, Santa Ros, 39-57, 3ª Planta, Esplugues de Llobregat, Barcelona 08950 Spain

**Keywords:** Code of ethics, Ethics, Mixed methods, Qualitative research, Questionnaire

## Abstract

**Background:**

The Catalan Institute of Health (CIH) is the largest health services public provider in Catalonia. “CIH Code of Ethics Virtual Forum” (CEVF), was created within the Intranet of the CIH to facilitate participation among their employees. The current study aims to: a) Analyse the CIH workers’ assessment of their own, their colleagues’ and the organization’s observance of ethical values; b) Identify the opinions, attitudes, experiences and practices related to the ethical values from the discourse of the workers that contributed voluntarily to the CEVF.

**Methods:**

Mixed methods study with **convergent parallel design**:

1. **Cross sectional study** by means of a self-administered, ad hoc, anonymous questionnaire to assess the observance of the ethical values of the CIH according to the participants. A total of 712 workers responded to the questionnaire. A descriptive, bivariate analysis of the results was carried out.
2. **Qualitative study** to determine the meaning for the workers of the ethical values put forward by the organization. Their individual opinions and experiences were explored by means of a thematic contents analysis of 225 comments posted in the CEVF.

The study was conducted between May and December 2008.

**Results:**

The average score for observance of the CE by the respondents themselves was high (over 4/5), between 3.5-4/5 for the observance by their colleagues and close to 3/5 for the CIH management. These results do not change when we compare by gender, age group and professional discipline. The comments on the values are information-rich, they mirror the ethical environment of the institution and show various ethical dilemmas and suggestions.

**Conclusions:**

Results show that it is feasible for a publicly funded health care organization to develop a CE with the participation of employees and the support of the management. Results underscore the relevance of this strategy for the implementation, improvement and update of the CE as a responsibility shared by all workers. Practices consistent with ethical values are rewarded by social approval, enhance employee’s confidence and coherence in decision-making and improve public engagement and institutional policies.

## Background

Health systems are continuously evolving due to new demographic and epidemiological conditions, technical and scientific advances, changes of expectations of society and social awareness of the capacity and rights of the public to make decisions about their health and the health services [1]. This process impacts on the relationship between health professionals and the public and the health system and society as a whole. It also needs to determine the relevant values that influence individual, collective and organizational decision making [2]. In essence, it is crucial that the workers share the values of the organization in order to conduct themselves in agreement with the corporate identity.

The relevance of a Code of Ethics (CE) is highlighted by the increasing number of institutions that have produced and implemented them during the last fifteen years [3–6]. Indeed, the CE is an important tool for the management of the social responsibility of institutions and the corporate covenant that determines the ethos that affects behaviour and decision making [4–6]. The CE must provide guidance and is a good instrument to present the values, concepts and ethical principles of the institutions. These values are the basis for the decisions taking to workers in front of ethical dilemmas It should be self-regulating and promote cooperation, dialogue and reflection and support the workers and the organization. Similarly to society, the CE will be in constant transformation and should be challenged and adapted to any situation.

The Catalan Institute of Health (CIH) is the largest health services public provider (primary medicine, specialists and hospital care) in Catalonia (Mediterranean region of Spain). It has a catchment population of almost six million people (75 % of all people with access to the publicly funded health system in Catalonia). In addition to health care provision, the CIH hospitals and primary care services undertake essential research responsibilities. In relation to higher education, the CIH trains 2,400 specialists in 49 different areas of the health sciences and over 4,500 medical, nursing, dentistry and other undergraduate students [7, 8]. It also includes non-clinical support personnel [9].

The CIH CE was the response to concerns voiced by management and professionals about the need for a charter that included the ethical values and behaviour regulations for all CIH personnel. The aim of the charter should be to strengthen the workers’ sense of responsibility and the public’s trust and to create an environment of shared values and involvement [10–12]. The CIH CE aims to promote behaviours of individuals and the organization in agreement with these ethical values. It does not intend to replace or interfere with the deontological codes of other professional groups [3]. Although the CE does not solve every ethical problem of an organization, it is an essential tool toward increasing ethical standards [6, 9], social legitimacy and improving coordination. Social legitimacy is particularly important for organizations with health provision responsibilities, where axiomatic, affective and cultural aspects are essential.

The CIH CE was written between 2000 and 2008. The project was conducted by the Code of Ethics Commission, with representation of all employees, external advisors and the management of the CIH. The management’s leadership and the workers’ participation were considered crucial. The first phase consisted of defining the corporate values. It was carried out by means of focal groups and questionnaires. During the drafting phase of values and behaviours a virtual space, the “CIH Code of Ethics Virtual Forum” (CEVF), was created within the Intranet of the CIH to facilitate participation. The objectives of the virtual forum were to provide a space of reflection and debate to solve and prevent ethical conflict, to collaborate with human resources to train workers in the implementation of the code and to advise them when faced with ethical issues. This strategy has been followed and recommended by some authors [13–21] and is also the focus of our analysis. A total of 6,815 CIH professionals participated in the process.

To understand the background and the moral conflicts that underpin clinical practice, it is essential to dig into the social construction of individual and collective ethical values of an organization [15, 16]. This study aims to: a) Analyse how individuals who work in the CHI evaluate observance of ethical values: their own, that of their colleagues and that of the organization as a whole; b) Identify opinions, attitudes, experiences and practices on ethical values as conveyed by the workers that contributed voluntarily to the CIH Code of Ethics Virtual Forum.

## Methods

### Design

Mixed methods study with a convergent parallel design that occurs when the researcher uses concurrent timing to implement the quantitative and qualitative strands during the same phase of the research process, prioritizes the methods equally and keeps the strands independent during the analysis [22]. It consisted of two phases: 1) Descriptive, cross sectional study by means of a questionnaire to assess the observance of corporate ethical values; 2) Qualitative study with a phenomenological approach to capture the meaning that employees of the CIH attribute to the ethical values put forward by the institution. Their own experiences in everyday practice were explored from the comments posted in the CEVF [23].

### Setting

The CE Commission set up the CEVF and encouraged the participation of all CIH workers.

### Dissemination strategy and recruitment of participants

We used several strategies of dissemination to encourage participation in the forum:

- A link and banner were added to the CIH intranet and the regional intranets of primary care and hospitals.
- Every time that information on the values was entered in the CEVF, an e-leaflet was sent to hospitals, primary care centres and laboratories of the CIH (During 2008, on March 4, April 23, May 29, September 18 and December 11).
- Two reminders in the page of electronic medical records (EMR).

Study period: May-December 2008.

### Quantitative Study: Questionnaire Code of Ethics Virtual Forum

A total of 712 CIH workers of different gender, age, qualifications, years of professional experience, work settings and geographical areas accessed the CEVF to respond to the questionnaire.

The CEVF questionnaire was self-administered, ad hoc and anonymous (Table 1). To asses the applicability and understandability of the questionnaire, a pilot of the questionnaire was carried out during April 2008 and the comments of 24 participants were taken into account. These 24 participants of the pilot study did not participated in the final sample of the study.

**Table 1:** Self-administered questionnaire completed by the workers of the Catalan Institute of Health in the Code of Ethics Virtual Forum. Catalonia, 2008

We want to know your opinion in relation to the current observance of our values in the workplace.			
*Score from 1 to 5: how do you think each value is observed* (1-This value is completely ignored; 5- This value is fully observed) by (YOU: in the context of work and responsibilities/YOUR COLLEAGUES: general assessment of the people in your team and your department/THE CIH : the institution, the managers).			
It will take you no more than 2 minutes to respond to this questionnaire.			
Value	You	Your colleagues	CIH
Competence	1-2-3-4-5	1-2-3-4-5	1-2-3-4-5
Respect	1-2-3-4-5	1-2-3-4-5	1-2-3-4-5
Responsibility/transparency	1-2-3-4-5	1-2-3-4-5	1-2-3-4-5
Confidentiality	1-2-3-4-5	1-2-3-4-5	1-2-3-4-5
Teamwork	1-2-3-4-5	1-2-3-4-5	1-2-3-4-5
Integrity	1-2-3-4-5	1-2-3-4-5	1-2-3-4-5
Equity	1-2-3-4-5	1-2-3-4-5	1-2-3-4-5
Quality	1-2-3-4-5	1-2-3-4-5	1-2-3-4-5

The questionnaire included 8 questions, one for each of the values considered (competence, respect, responsibility and transparency, confidentiality, teamwork, integrity, equity and quality). Each value was given a value from 1 to 5 in a Likert-type scale, with verbal anchors located at each end (1: this value is completely ignored; 5: this value is fully observed). Participants entered their opinion on their own observance of these values, the observance by colleagues (people in their team or department) and by CIH management. In order to access the CEVF and to respond to the questionnaire, they had to enter the following data: age group (≤30 years, 31–50 and > 50 years), gender, years of professional experience in the CIH (≤10 and >10 years), professional discipline (nurse, doctor, social worker, other health workers, administrative personnel and janitors) and code (user name chosen by the participant).

### Statistical analysis

A descriptive, bivariate analysis was carried out with the answers to the questionnaire. Student’s *t* and analysis of variance were used for independent data and Friedman’s test for relational data. The statistical significant level was set at p ≤0.05 for two-sided hypotheses. Analysis was carried out with the statistical package SPSS 18.0.

### Qualitative study: Open Comments in the Code of Ethics Virtual Forum

#### Data collection techniques

The CE Committee created a document with suggestions on the definition and the behaviours to encourage and to avoid in relation to each of the values. They also presented some cases to illustrate everyday situations that pose ethical challenges (Table 2). Based on these cases, participants wrote about their experiences and opinions about the CE values and ethics within the institution. The CE Committee moderated and responded to questions and thoughts of the participants when necessary.

**Table 2:** Case reports and answers to the values considered in the Code of Ethics Virtual Forum of the Catalan Institute of Health. Catalonia, 2008

Value	Summary of the case report of the forum^a^	Possible answers^a^
Competence Capacity and continuing professional development to work to the best of your ability.	A worker with 20 years of experience spots in the intranet a course on multiculturality and migrations for people with her training and during working hours.	a) Dismisses the issue since she considers she is experienced enough and does her job well. b) She asks her manager for leave to do the course. c) She is aware that it is an emerging issue and asks to participate.
Respect Attitude of consideration and care for the patients and their sense of dignity.	You are visiting a patient when a colleague comes and interrupts and starts talking to you; he/she has something to ask you.	a) You listen to your colleague and answer the question, you know it is not the right thing for the patient but it is sort of customary. b) You listen to your colleague, answer the question and when he/she leaves you complain to the patient and apologize. c) You listen to your colleague, explain that you are with a patient and that if it’s not an emergency you’ll get back to him/her. d)You ask the patient if you may answer the question and explain that is it your colleague.
Responsibility Capacity to assume our own decisions and their consequences and to explain them to the people involved.	A patient comes on Sunday at 14.30 when everybody on call is having lunch together.	a) I make the patient wait without explaining until we finish lunch. b) I ask if he/she can wait until we finish. c) I explain that the doctor is busy with an emergency and that he/she will have to wait. d) I ask the patient if he/she can wait, that we are finishing lunch and will be there in 10 minutes. I add that if there is any problem we’ll see him/her immediately.
Confidentiality Moral duty of secrecy and to make a limited and well defined use of the information we hold on the patients.	A relative requests information about your patient in the corridor of your health centre.	a) Tell him or her in the corridor that you cannot provide information because of confidentiality. b) You provide the information. c) You go to an office and provide the requested information. d) You go to an office and explain that you cannot give that information.
Teamwork Coordination of professionals to provide services to patients and the community.	An urgent meeting is scheduled in your department first thing in the morning. When the meeting starts you are still visiting patients.	a) You don’t go (it’s not that important) and finish your clinical work. b) You finish the clinics and go to the meeting, even if it’s late. c) You reorganise your clinical work and go to the meeting pointing out the inconvenience of such short notice. d) You reorganise your appointments first thing in the morning to have enough time to arrive to the meeting when it starts. e) You leave the patients in the waiting room and attend the meeting.
Integrity Capacity to make decisions, to act and to respond of your own behaviour in accordance with professional values.	You meet a pharmaceutical representative in the hospital/primary care centre. He/she invites you to the next congress of your specialty and he/she reminds you of the medicines of his/her pharmaceutical company.	a) I accept, I don’t think this will influence my prescribing. b) I accept, I assume that it influences my prescribing but I’m really interested in this congress. c) I refuse, I understand that I have to pay for my expenses. I would like my department to generally agree to some funding for meetings and training.
Equity Dealing with the circumstances and values of the people fairly without discrimination.	A department in a hospital gets new devices when another department lacks many basics.	a) Inequities must be explained. If resources are prioritized it must be explained. b) The management must avoid another similar situation in the future. c) Management must undertake to improve the worse-off departments.
Innovation Introduction of changes for improvement taking into account risk/benefit.	A health centre invests in new technological devices that are not used for months.	a)There are other priorities and a high healthcare workload. The use of the new devices is postponed. b)The manager of the health centre decides for the devices to be used without training thus limiting the services that they are able to provide. c)The manager of the health centre organises training to optimise use of resources.

^a^The case reports and possible answers were prepared by the Code of Ethics Commission. They constituted the basis for the comments of the participants and for the qualitative study

A total of 225 comments were posted in reply to the illustrative cases on challenging ethical situations related to each value. Participants could add behaviour to be encouraged and to be avoided (Table 2).

#### Analysis

A descriptive-interpretive thematic contents analysis of the comments posted in the forum was carried out each time that a different value was introduced. The text of the analysis were these same comments [24]. The analysis consisted of: a) careful reading of all comments; b) identification of relevant topics and texts; c) fragmentation of the texts in units of meaning; d) codification of texts by means of emerging codes; e) determination of clusters of codes by analogy with the objectives of the study; f) identification of first-order emerging categories; g) determination of second-order categories based on the ethical values of the CIH and; g) triangulation of results amongst three members of the research team. Analysis was carried out manually and with the support of Atlas.ti.

#### Ethics

The study follows the Primary Care Research Good Practice Guidelines of the IDIAP Jordi Gol [25] and was approved by the Clinical Research Ethics Committee of IDIAP Jordi Gol (P15/029). The study follows the Primary Care Research Good Practice Guidelines of the IDIAP Jordi Gol [25] and was approved by the Clinical Research Ethics Committee of IDIAP Jordi Gol (P15/029). The participant was registered voluntarily in the Virtual Forum of code Ethics, giving their user name (anonimized), type of job, gender and age. To guaranty confidentiality and anonymity, they chose an username. With this user name, the participant accessed to the survey once, and they can make comments to the forum as much as they wanted. They did not sign a consent form expressly, but with their voluntary participation in the forum, we could assume that they gave the verbal consent. Moreover, the username blind their personal identification.

## Results

Between May and December 2008 a total of 4,640 professionals from different areas of Catalonia and different professional disciplines registered in the CEVF: 75 % (*n* = 3,475) worked in the corporate or regional headquarters; 7.5 % (*n* = 335) in hospitals and 18 % (*n* = 830) in primary care. The questionnaire was completed by 712 people and 225 comments were posted in the CEVF.

### Results

#### Quantitative study: Questionnaire on the values of the Code of Ethics

Table 3 shows the results of the self-administered questionnaire. Significant differences were obtained for all values (*p* < 0.001) between the self-evaluation (observance of the value by the worker him/herself) and the assessment of their colleagues and the CIH. The average scores were over 4 for the self-assessment of observance of ethical values, between 3.5-4 for the assessment of the colleagues and close to 3 for the assessment of the CIH.

**Table 3:** Results of the self-administered questionnaire

*n* =712	1 point	2 points	3 points	4 points	5 points	Mean (SD)^a^ score	p-Value**
	n (%)	n (%)	n (%)	n (%)	n (%)		
Competence							
*You*	3 (0.4)	6 (0.8)	74 (10.4)	348 (48.9)	281 (39.5)	4.3 (0.7)	<0,001
*Your colleagues*	9 (1.3)	40 (5.6)	226 (31.7)	311 (43.7)	126 (17.7)	3.7 (0.9)	
*THE CIH*	49 (6.9)	161 (22.6)	266 (37.4)	169 (23.7)	67 (9.4)	3.1 (1.1)	
Respect							
*You*	2 (0.3)	3 (0.4)	44 (6.2)	322 (45.2)	341 (47.9)	4.4 (0.7)	<0,001
*Your colleagues*	91.3	344.8	18425.8	31844.7	16723.5	3.8 (0.9)	
*THE CIH*	56 (7.9)	126 (17.7)	257 (36.1)	191 (26.8)	82 (11.5)	3.2 (1.1)	
Responsibility/Transparency							
*You*	0	2 (0.3)	52 (7.3)	319 (44.8)	339 (47.6)	4.4 (0.6)	<0,001
*Your colleagues*	10 (1.4)	26 (3.7)	191 (26.8)	307 (43.1)	178 (25.0)	3.9 (0.9)	
*THE CIH*	75 (10.5)	147 (20.6)	225 (31.6)	183 (25.7)	82 (11.5)	3.1 (1.2)	
Confidentiality							
*You*	1 (0.1)	8 (1.1)	71 (10.0)	224 (31.5)	408 (57.3)	4.5 (0.7)	<0,001
*Your colleagues*	12 (1.7)	43 (6.0)	157 (22.1)	226 (31.7)	274 (38.5)	4.0 (1.0)	
*THE CIH*	25 (3.5)	85 (11.9)	132 (18.5)	216 (30.3)	254 (35.7)	3.8 (1.1)	
Teamwork							
*You*	6 (0.8)	21 (2.9)	115 (16.2)	308 (43.3)	262 (36.8)	4.1 (0.8)	<0,001
*Your colleagues*	22( 3.1)	81 (11.4) )	224 (31.5)	258 (36.2)	127 (17.8)	3.5 (1.0)	
*THE CIH*	90 (12.6)	143 (20.1	249 (35.0)	152 (21.3)	78 (11.0)	3.0 (1.2)	
Integrity							
*You*	2 (0.3)	7 (1.0) )	72 (10.1)	333 (46.8)	298 (41.9)	4.3 (0.7)	<0,001
*Your colleagues*	11 (1.5)	44 (6.2	187 (26.3)	312 (43.8)	158 (22.2)	3.8 (0.9)	
*THE CIH*	63 (8.8)	133 (18.7)	238 (33.4)	186 (26.1)	92 (12.9)	3.2 (1.1)	
Equity							
*You*	0	6 (0.8)	70 (9.8)	329 (46.2)	307 (43.1)	4.3 (0.7)	<0,001
*Your colleagues*	12 (1.7)	34 (4.8)	146 (20.5)	334 (6.9)	186 (26.1)	3.9 (0.9)	
*THE CIH*	72 (10.1)	119 (16.7)	172 (24.2)	205 (28.8)	144 (20.2)	3.0 (1.3)	
Quality							
*You*	1 (0.1)	16 (2.2)	103 (14.5)	358 (50.3)	234 (32.9)	4.1 (0.7)	<0,001
*Your colleagues*	9 (1.3)	38 (5.3)	189 (26.5)	325 (45.6)	151 (21.2)	3.8 (0.9)	
*THE CIH*	62 (8.7)	117 (16.4)	235 (33.0)	199 (27.9)	99 (13.9)	3.2 (1.1)	

Scale of assessment: from 1 to 5 (1-no observance of this value, 5-complete observance of this value).

*CIH Catalan* Institute of Health; SD^a^ = Standard deviation; **p value calculated according to the analysis of variance to compare independent means

Comparisons based on gender, age groups, years of professional experience and professional discipline were similar to the results previously described (Table 4). Significant differences were found between genders in the score for the CIH, where women gave higher scores for the values Competence, Responsibility/transparency, Teamwork, Integrity and Quality. In the analysis by age groups, professionals under 30 years of age gave higher scores to the value Integrity to their colleagues and Equity to the CIH. In relation to years of professional experience (up to 10 years and 11 years and over), results were very similar; however, those professionals with less years of experience gave higher scores to the observance of values by the CIH with statistically significant differences in the values Confidentiality, Integrity and Quality. Results were also very similar in the comparison by professional discipline, though the social workers (*n* = 10) gave higher scores to the CIH. In contrast, physicians gave lower scores to the CIH, with significant differences in the Respect, Responsibility/Transparency, Confidentiality, Teamwork and Integrity values (Table 5).

**Table 4:** Results of the workers’ self-administered questionnaire in the Code of Ethics Virtual Forum of the Catalan Institute of Health

	Gender (*n* = 577)		Age groups (*n* = 450)			Years of professional experience (*n* = 456)	
	Men	Women	≤30 years	31-50 years	>50 years	≤10 years	>10 years
	Mean (SD)* (*n* = 182)	Mean (SD)* (*n* = 385)	Mean (SD)* (*n* = 19)	Mean (SD)* (*n* = 245)	Mean (SD)* (*n* = 186)	Mean (SD)* (*n* = 115)	Mean (SD)* (*n* = 341)
Competence							
*You*	4.2 (0.7)	4.3 (0.7)*	4.4 (0.7)	4.2 (0.7)	4.3 (0.7)	4.3 (0.7)	4.2 (0.7)
*Your colleagues*	3.7 (0.9)	3.7 (0.9)	3.9 (0.9)	3.7 (0.8)	3.7 (0.9)	3.7 (0.9)	3.7 (0.8)
*THE CIH*	2.8 (1.1)	3.2 (1.0)**	3.3 (0.9)	3.0 (1.0)	3.0 (1.1)	3.1 (1.0)	3.0 (1.1)
Respect							
*You*	4.3 (0.6)	4.4 (0.7)	4.3 (0.8)	4.4 (0.6)	4.4 (0.7)	4.3 (0.6)	4.4 (0.7)
*Your colleagues*	3.8 (0.8)	3.9 (0.9)	4.0 (0.9)	3.9 (0.8)	3.8 (0.9)	3.8 (1.0)	3.9 (0.8)
*THE CIH*	3.1 (1.1)	3.2 (1.1)	3.6 (0.8)	3.2 (1.1)	3.1 (1.1)	3.3 (1.0)	3.1 (1.1)
Responsibility/Transparency							
*You*	4.3 (0.7)	4.4 (0.6)	4.3 (0.7)	4.4 (0.6)	4.4 (0.7)	4.4 (0.6)	4.4 (0.6)
*Your colleagues*	3.8 (0.8)	3.9 (0.9)	3.8 (0.8)	3.8 (0.9)	3.9 (1.0)	3.8 (0.9)	3.9 (0.9)
*THE CIH*	2.8 (1.2)	3.2 (1.1)**	3.4 (1.0)	3.0 (1.1)	3.1 (1.1)	3.2 (1.1)	3.0 (1.1)
Confidentiality							
*You*	4.4 (0.7)	4.4 (0.8)	4.4 (0.8)	4.3 (0.8)	4.5 (0.7)	4.4 (0.8)	4.4 (0.7)
*Your colleagues*	3.9 (1.0)	4.0 (1.1)	4.0 (1.2)	3.9 (1.0)	4.0 (1.1)	3.9 (1.1)	3.9 (1.0)
*THE CIH*	3.6 (1.3)	3.9 (1.1)	4.1 (0.9)	3.8 (1.1)	3.7 (1.2)	4.0 (1.0)	3.7 (1.2)*
Teamwork							
*You*	4.0 (0.9)	4.2 (0.8)*	4.4 (0.6)	4.1 (0.9)	4.1 (0.9)	4.2 (0.9)	4.1 (0.9)
*Your colleagues*	3.4 (1.1)	3.6 (1.0)*	3.6 (1.2)	3.5 (0.9)	3.5 (1.1)	3.5 (1.1)	3.5 (1.0)
*THE CIH*	2.8 (1.3)	3.1 (1.1)*	3.0 (1.1)	2.9 (1.1)	2.9 (1.2)	3.0 (1.1)	2.9 (1.2)
Integrity							
*You*	4.2 (0.7)	4.3 (0.7)	4.6 (0.5)	4.2 (0.7)	4.2 (0.8)	4.3 (0.7)	4.2 (0.8)
*Your colleagues*	3.7 (0.9)	3.8 (0.9)	4.3 (0.8)*	3.8 (0.9)*	3.7 (1.0)*	3.8 (1.0)	3.7 (0.9)
*THE CIH*	3.0 (1.2)	3.2 (1.1)*	3.6 (0.8)	3.1 (1.1)	3.1 (1.2)	3.3 (1.1)	3.1 (1.1)*
Equity							
*You*	4.3 (0.7)	4.3 (0.7)	4.5 (0.7)	4.3 (0.7)	4.2 (0.7)	4.3 (0.7)	4.3 (0.7)
*Your colleagues*	3.9 (0.9)	3.9 (0.9)	4.1 (1.1)	3.9 (0.9)	3.9 (0.9)	3.9 (1.0)	3.9 (0.9)
*THE CIH*	3.2 (1.3)	3.4 (1.2)	4.0 (1.0)*	3.3 (1.3)*	3.2 (1.2)	3.5 (1.2)	3.2 (1.2)
Quality							
*You*	4.1 (0.8)	4.1 (0.8)	4.3 (0.8)	4.1 (0.8)	4.1 (0.8)	4.1 (0.8)	4.1 (0.8)
*Your colleagues*	3.7 (0.9)	3.8 (0.9)*	4.1 (1.0)	3.7 (0.8)	3.8 (0.9)	3.8 (0.9)	3.8 (0.9)
*THE CIH*	3.0 (1.2)	3.3 (1.1)**	3.7 (1.1)	3.1 (1.1)	3.2 (1.2)	3.4 (1.1)	3.1 (1.1)*

Scale of assessment: from 1 to 5 (1-no observance of this value, 5-complete observance of this value). CIH = _Catalan Institute of Health; SD = Standard deviation.*p < 0.05; **P < 0.001

**Table 5:** Results of the workers’ self-administered questionnaire in the Code of Ethics Virtual Forum of the Catalan Institute of Health according professional disciplines

*n* =456	Nurses	General proctitioners	Social Workers	Other health workers	Auxiliaries and administratives	P value
	Mean (SD) (*n* = 131)	Mean (SD) (*n* = 173)	Mean (SE) (*n* = 10)	Mean (SD) (*n* = 121)	Mean (DE) (*n* = 121)	
Competence						
You	4,3 (0,7)	4,2 (0,7)	4,2 (1,0)	4,3 (0,7)	4,3 (0,8)	0,810
Your colleagues	3,7 (0,9)	3,8 (0,8)	3,9 (1,0)	4,0 (0,8)	3,6 (0,9)	0,161
THE CIH	3,1 (1,1)	2,9 (1,0)	3,3 (1,4)	3,2 (0,9)	3,1 (1,1)	0,404
Respect						
You	4,3 (0,6)	4,4 (0,7)	4,7 (0,7)	4,3 (0,6)	4,4 (0,6)	0,342
Your colleagues	3,8 (0,9)	3,9 (0,8)	4,2 (0,8)	3,7 (0,9)	3,9 (0,9)	0,652
THE CIH	3,2 (1,1)	2,9 (1,1)*	3,5 (1,1)	3,3 (1,1)	3,3 (1,1)*	0,013
Responsibility/Transparency						
You	4,3 (0,7)	4,3 (0,6)	4,2 (1,0)	4,5 (0,6)	4,5 (0,6)	0,073
Your colleagues	3,9 (0,9)	3,9 (1,0)	3,8 (0,8)	4,1 (0,7)	3,9 (1,0)	0,848
THE CIH	3,2 (1,1)*	2,8 (1,1)*	3,1 (1,3)	3,3 (1,1)	3,1 (1,1)	0,032
Confidentiality						
You	4,4 (0,7)	4,3 (0,8)*	4,2 (0,8)	4,5 (0,6)	4,6 (0,6)*	0,004
Your colleagues	3,9 (1,0)	3,8 (1,0)	4,0 (0,8)	4,2 (1,0)	4,1 (1,1)	0,168
THE CIH	3,8 (1,0)*	3,4 (1,2)*	3,9 (0,9)	4,3 (0,9)*	4,0 (1,1)*	<0,001
Teamwork						
You	4,1 (0,9)	3,9 (0,9)*	4,1 (1,2)	3,9 (0,9)	4,3 (0,7)*	0,005
Your colleagues	3,5 (1,1)	3,5 (0,9)	4,0 (1,2)	3,4 (0,9)	3,5 (1,1)	0,596
THE CIH	3,0 (1,1)	2,7 (1,1)*	3,3 (1,3)	3,2 (0,9)	3,2 (1,1)*	0,003
Integrity						
You	4,2 (0,7)	4,1 (0,8)*	4,3 (0,8)	4,2 (0,6)	4,4 (0,7)*	0,028
Your colleagues	3,8 (0,9)	3,7 (0,9)	3,9 (1,0)	3,6 (1,0)	3,8 (1,0)	0,900
THE CIH	3,1 (1,1)	2,9 (1,1)*	3,5 (1,3)	3,4 (1,0)	3,3 (1,1)*	0,009
Equity						
You	4,3 (0,8)	4,2 (0,7)	4,2 (0,9)	4,3 (0,6)	4,4 (0,6)	0,282
Your colleagues	3,9 (0,9)	3,9 (0,9)	3,6 (0,7)	3,9 (0,8)	3,9 (0,9)	0,844
THE CIH	3,2 (1,3)	3,2 (1,3)	3,6 (1,3)	3,4 (1,1)	3,5 (1,2)	0,208
Quality						
You	4,2 (0,8)	4,0 (0,8)*	3,8 (0,9)	4,1 (0,5)	4,2 (0,9)*	0,034
Your colleagues	3,8 (0,9)	3,7 (0,9)	3,8 (0,9)	3,8 (0,8)	3,8 (0,9)	0,881
THE CIH	3,2 (1,1)	3,0 (1,1)	3,6 (1,0)	3,3 (1,1)	3,4 (1,2)	0,070

Scale of assessment: from 1 to 5 (1-no observance of this value, 5-complete observance of this value). CIH = Catalan Institute of Health; SD* = Standard deviation; *p < 0.05

**Table 6:** Analysis of the Value “Confidentiality”. Worker’s comments in the Code of Ethics

Category 1. Institutional, organizational and management aspects	
Aspects in need of improvement	Suggestions
• The management of space is prioritized when building health centres. • Operability is prioritized over confidentiality. • To solve high workloads, decisions are made that conflict with confidentiality, for instance: • Signature of prescriptions by administrative personnel. • Administrative personnel triage the most urgent cases.	• To sign an individual confidentiality form for access to the information necessary to provide healthcare. • To define and disseminate the levels of access to Electronic Medical Records. • To define functions and responsibilities of employees. • To analyse infringements of confidentiality. • To assume responsibility in situations of infringement of confidentiality. • Strategies to request referrals and tests in agreement with confidentiality requirements.
Category 2. Facilities	
Aspects in need of improvement	Suggestions
• Low quality of building in healthcare facilities, even in the most recent. The management of space is prioritized when building health centres. • Rooms are not soundproof. • In the waiting rooms and doctors’ offices you can listen to the conversations of professionals and patients. • The lack of an office to give confidential information is commonplace.	• To guarantee that the new facilities comply with confidentiality requirements. • To design the interior of health centres to comply with confidentiality requirements. Separate entrances for users and personnel. • Exclusive areas for personnel.
Category 3. Confidentiality , Information Systems and Electronic Medical Records	
Aspects in need of improvement	Suggestions
• Confidentiality gets lost when information is shared with other professionals. • Everybody can access the Electronic Medical Records. • The current medical records do not guarantee confidentiality. • Electronical medical records are very useful, but I’m not sure what happens then with confidentiality. • Many people has access to the information on referrals. • The patient might feel unprotected.	• To open clinical records only with the agreement of the patient. • To define and disseminate levels of access to the Electronic Medical Records. • To sign an individual confidentiality form for access to the information necessary to provide healthcare. • To register when the clinical records are accessed. • To improve confidentiality in the management of documents with clinical data.
Category 4. Training and raising awareness on confidentiality for health workers and society at large	
Problems identified	Suggestions
• There is no training on confidentiality. • Errors occur due to lack of knowledge.	• To inform workers on the legal implications of violating confidentiality. • Training in schools and universities and for all CIH professionals. • Campaigns of training and social awareness on confidentiality.
Category 5. Confidentiality as a cross professional competence in the CIH	
Aspects in need of improvement	Suggestions
• Confidentiality is not observed. • We hear comments on patients and colleagues in corridors. • Confidentiality is violated during informal conversations between colleagues. • There is gossiping on confidential information. • Some professionals access more information than they require. • A brief medical history is taken at the entrance of A&E. Information, even about severe diagnostics, is sometimes delivered along corridors. • Relatives are given information at the door of the ward.	• Be clear on behaviours that violate confidentiality. • Improve attitudes and manners. • Do not give information on corridors or places that do not provide any privacy. • Do not give information by phone. • Inform the patient and ask if it is ok to have residents or medical students during the visit. • Leave time for the patient-doctor relationship during ward rounds.
General opinions	Positive experiences
• Confidentiality is a problem of difficult solution. • It is an unresolved issue. • It is everybody’s right and responsibility. • Confidentiality is the responsibility of professionals. • Information should be given in private, with enough time for the patient, relatives and the professional in charge to voice their concerns. • Confidentiality has limitations when individual rights are in conflict with social rights.	• I look for those areas in the waiting room that guarantee confidentiality. • I do not raise my voice. • Family members generally accept that I cannot give information to them.

#### Qualitative study: Comments of the Code of Ethics Virtual Forum

Results of the analysis of 225 comments clustered by values of the CE (second-order, predefined categories), emerging categories and the most relevant verbatims are shown.

This 225 comments correspond to 191 different people (1 person made 13 comments; 2 people made 4 comments, 1 person made 3 comments and 5 people made 2 comments). Besides four comments were accurate responses to inquiries CCE.

##### Value competence

There was an only comment on this value (Table 2), which urged a careful reading and a reflection on the definition of this value. In particular, the participant admitted to behaviour to be avoided.

*“…. I, today, admit to some behaviour that we better avoid. I have done that on occasion. Read it and take time to think truthfully. I think that we / they… need it”.*

##### Value respect

The participants scored the value respect very highly. We can extract three categories from their comments:

- Respect to patients
  Based on the illustrative case, a participant explains that the interruptions in the space of relationship with the patients are very frequent. These interruptions take place during consultations and are usually caused by the professionals, though not always. The comments show great diversity of interruptions: from waiting for a pause in the conversation to ask something to being plainly rude.
    *“I try to show respect at all times, but I see my colleagues that lose it very easily… the same goes for the users of the health services”.*
- Respect for the choices and decisions of patients and their families
  A participant explains his/her own experience on the care of a family member with a terminal disease that shows the lack of respect for the preferences and decisions of patients and their families. While this is only one case, the participant adds that some of his/her colleagues have experienced similar situations.
    *“… as CIH professionals, we all should have a very clear picture of what is the respect to a terminal patient. A year ago I suffered the bitter experience of having my terminal father admitted to a big CIH hospital. I almost had to demand for sedation since the personnel there only gave him paracetamol. Why don’t we accept that the moment to die arrives and that the patient and his/her family only want this transition moment to be as quick and painless as possible?”.*
- Respect toward the job and decisions of the professionals
  Participants highlight the key role of the organization in relation to the respect observed for the workers, the need to acknowledge a job well done and to respect professional and academic skills with equity.
    *“To value more a certain type of professional…; to score higher because of stress …; to give more points for a language than for a PhD…. People do not feel more appreciated because they are paid more than others with the same training. I’m not saying you should not pay proportional salary increases or for training time”.*

##### Value responsibility and transparency

The following categories emerged:

- Answers and comments of the participants on the illustrative cases
  The messages about the case (Table 2) try to find a balance between responding to patients’ needs and taking care of themselves as professionals.
    *“… if I think it is fine to wait I will ask the patient to wait, but I would not explain the cause of the delay, I really do not have to explain that. The patient has a right to care and to be informed, but there is no need to get into internal matters”.*
- Interference of computers in the professional-patient relationship
  Patients often comment on the interference of the computer during visits and its effects: feeling of not being listened to, decreased trust in the treatment and higher attendance.
    *“It is a comment that you frequently hear amongst patients, it is very annoying and the patients leave feeling that the doctors have not paid attention to them. In consequence, they do not trust that the treatment they have been prescribed is correct and tend to come back more often”.*
- Poor phone access
  Some patients complain that the telephone lines are never available and they spend a lot of time trying to contact the health services. This does not reflect well on the organization and should be solved.
    *“What’s the use of a phone service that does never work? As always, those who suffer are the patients that have tried for days to communicate via phone with the health centre, and need eventually to come to get the appointment …”*

##### Value Confidentiality

The highest number of comments in the CEVF (*n* = 31) were posted for the value confidentiality. The following four categories emerge from the analysis of these comments (Table 6):

- Institutional and management aspects
  The debate shows the inconsistencies between the legal framework and reality in relation to confidentiality matters. Participants explain that confidentiality is infringed on a daily basis, in A&E (for instance, informing patients and relatives in corridors or at the door of hospital wards) and also in the reception desks of primary care centres (where triage of the most urgent cases is often carried out). The facilities often go against the right to confidentiality.
    *“Manners and attitudes must improve, professionals need to understand the basics. It is really sad to talk about intimate and crucial aspects of the life of your father in the middle of a corridor because the doctor is “in a hurry”, while people come and go, just as it happened to me”.*
- Facilities
  Some workers think that the health services facilities, even the most modern, do not safeguard the privacy of patients. There are no purposely designated rooms to inform the patients and in some doctors’ offices everything can be heard even when the door is closed. In shared rooms in hospitals patients know about the conditions of their room companions. Other professionals add that even if there are no purpose-built rooms to this end, a more private location can usually be found.
    *“Hi, while I write, I can listen to the conversation of my colleague in the office next to mine. Even though both doors are closed, I could truly write word by word everything he says. I work in a 5-month old facility and you can see that it already infringes on the principle of confidentiality. The people in charge of facilities should do a better job. Could I add that the quality of building of these facilities is appalling? We would not tolerate this in our homes. Why, then, are we allowing it to happen in a public building paid by all of us?”*
- Information systems, Electronic Medical Records
  Health workers worry about the impact of electronic medical records on confidentiality. Although most consider EMR an advance, they wonder about sharing it with other health centres and question if information that has been given to a specific health professional in the context of mutual trust should be shared at all. Some even choose not to register particular information. Other professionals explain the problem of not being able to access important information in case of emergency and the difficulty of remembering the information that is not recorded in the medical records. Breaks of confidentiality in relation to referrals are also mentioned, though professionals admit that such breaks of confidentiality did also happen with traditional medical records on paper.
    *“Since a experience I had, I never write in the electronic medical records about diseases or conditions that have some stigma attached (sexually transmitted diseases, AIDS, abortion)…. the trust of the patient is with his/her doctor and not with every doctor in Catalonia, even when we take into account professional secrecy....”*
- Attitudes, behaviours, training and awareness of CIH workers and of society as a whole.
  Participants highlight attitudes and behaviour of employees in relation to confidentiality and they point out at the need for training to raise awareness. They mention some courses on this matter, although they are perceived as insufficient. Participants explain that confidentiality infringement can be caused by lack of knowledge or by attitudes and behaviours of workers for whom this value is not important.
    *“I think that there is lack of training on this essential topic. Mistakes are made with good intentions, such as when you give information to some relatives… we all have a right to confidentiality and moreover, we should be fierce custodians of this right”.*
    *“Confidentiality is still unresolved in the hospitals, comments about patients and colleagues in the corridors are commonplace”.*
  Table 5 shows the contributions of participants on confidentiality listed by emerging categories, aspects in need of improvement and suggestions.

##### Value teamwork

Three categories on this value emerge from the analysis of the comments posted:

- Questions, answers and opinions on the illustrative case
  Some participants explain that this case (Table 2) is about time management and managers’ lack of planning. They question the meaning of “urgent meeting” and the real need for a meeting that requires delaying clinical appointments. Some participants respond that if the meeting is indeed urgent and important they should attend, but not before rescheduling and taking care of the patients.
    *“… Can a manager say that a meeting is urgent? Is there any cause for a meeting that obstructs clinical practice? I rather think that it is bad management. Managers often forget that they also have moral obligations, since they belong to the human species. For me, the best option is to plan the visits and the meeting properly”.*
- Attitudes and opinions of participants on teamwork
  Participants have a positive attitude toward teamwork. They consider that lack of teamwork has a negative impact on the team and the patient.
    *“We are all necessary within health services, patients need integrated care, therefore we all have our own particular role and a common role…”.*
- Barriers to teamwork
  With respect to teamwork, we identified barriers related to attitudes and behaviours of workers and of the organization. The workers themselves point to tensions, estrangement, hierarchy and class prejudices (in particular coming from workers with higher education), and lack of respect toward colleagues. Other barriers are lack of knowledge and communication between colleagues and the absence of a culture of teamwork and common purpose.
    *“…Some employees are totally nasty with those of a lower category and very cynical with their own work. All that…. makes me very sceptical about teamwork. There is no team. All of us, from the cleaners to the most important doctor, we all deserve respect. If the concept of team fails, the work and the patients will suffer the consequences”.*
  In relation to barriers to teamwork that originate from the organization, some management initiatives such as the dismantling of the Health Care Basic Units or the demarcation of functions by professional category have contributed to increase antagonism between professional disciplines, the perception of overload and competence. Participants highlight the lack of initiatives coming from the organization to support teamwork.
    *“Since legal responsibility is individual and the incentives are a unilateral imposition and propitiated by financial lure, the necessary conditions for teamwork do not exist …”.*
  A participant explains that some labels used by human resources such as “professional category” and “class” are old fashioned and tend to divide professionals.

##### Value integrity

Three categories were extracted from the analysis of the comments:

- Integrity in relation with the pharmaceutical industry
  One participant mentions the complicity of some employees with the pharmaceutical industry. She considers this behaviour inacceptable. *“I do not see many comments on this subject. I’m not surprised, since we take part in a market with the pharmaceutical industry. To me, this is unacceptable, but nobody (not even the management) wants to get rid of it.”*
  Some participants comment on the presence of objects with publicity from the pharmaceutical industry such as pens in doctors’ offices. While this could be considered rather innocent, it could also lead to a higher prescription of medicines better known to patients. These participants insist on the absence of advertising in public health facilities.
- Attitudes and behaviours of participants
  Some participants point out that presents should not be accepted under any circumstance. They show integrity and the value of independence.
    *“It is a difficult issue. Now, in my office, no brand can be seen and I use the pens and stickers of the CIH, they work. I don’t think I’ll ever accept funds to attend a medical meeting. I consider it inexcusable”.*
- Integrity and incentives to achieve the objectives of variable productivity
  The illustrative case promotes reflection on integrity in relation to achievement of objectives and incentives based on variable productivity. The participant points out that some objectives should be achieved because they are what constitutes “a job well done, which has a big impact on health”, independently of incentives. The example explains the objective to achieve exclusive breastfeeding during the first 6 months of life, which means to care for the health of the babies and for the economy of their families. The comments suggest that in some cases the professionals prioritize achievement of objectives and incentives.

##### Value equity

The following categories emerge from the analysis of cases:

- Equity in the management of materials and human resources
  One participant reflects on the ethics of offering incentives to health professionals, and believes that some strategies such as management by objectives and to obtain good results in professional advancement might be contrary to the deontological code.
    *“What is the ethics of a public organisation that promotes on purpose falsification of information –by means of the objectives and professional advancement? They do not provide supply workers during times of high workload and replace doctors and nurses to manipulate statistics, to rearrange the registers of the management by objectives”.*
  In relation to the case of lack of equity in the distribution of resources:
    *“We do not have our own budget nor allocated posts; … the employees have temporary contracts or contracts for just a month, with all the uncertainty it entails…”.*
- Equity in access and coverage of health services
  Participants question equity in accessibility and universal coverage of our health system. They offer examples of access inequity (limited coverage to patients from other Spanish autonomous communities, third party billing and lack of compensation) and pose questions on specific areas. These questions are evaluated by the Primary Care Clinical Ethics Committee of the IDIAP Jordi Gol, which provides answers based on the current regulations on health coverage when moving from Catalonia to another EU country, to a non-EU country with bilateral agreement and to a non-EU country without bilateral agreement.
    *“I’d like to know the opinion of the Code of Ethics Commission on the situation of a Catalan worker who moved abroad but who pays the Spanish National Insurance and who is denied compensation of his expenses for being in a country without health services agreement. In contrast, we have a non-Spanish patient that does not work nor live in Spain and comes every 2 months to get a treatment paid by the CIH (20.000 euro/year)”.*
- Ethical dilemmas on childhood immunizations
  One participant explained the ethical dilemma brought about by the lack of agreement within a paediatric team. The question was about the obligation to inform parents about the vaccines that are not included in the official immunization schedule and need to be paid for (pneumococcal, rotavirus and varicella vaccines). This situation was encountered in a primary care team with a catchment population of a low socioeconomic level, a high rate of immigrants, and taking into account that these vaccines are expensive.
    *“Some believe that it is ethical to inform despite the population we attend (imagine explaining all these to some immigrants!). Some believe that informing is unethical because you cause anxiety in the family and they think that these vaccines are not efficient (also because it is also the belief of the administration) and for these families the cost must especially be taken into account”.*
  The Clinical Ethics Committee tried to determine the optimal response to this dilemma. The answer started with a reflection on the right to information (autonomy of the user, in this case represented by the parents) and the principle of justice in the management of health resources.
    *“The physician is under the ethical obligation to inform the patient of the official immunization schedule and must try to implement it to guarantee the recommendations of the health authorities for a good public health standard”.*

##### Value innovation

During the study period no comment on the value innovation was posted on the CEVF.

## Discussion and conclusions

This mixed methods study shows the participants’ assessment of the observance of the CIH CE and the opinions, attitudes, experiences, needs and practices of the employees in relation to these values. The methodological strategy offers a polyhedric perspective which is relevant and applicable to the subject under study [26].

The results of the **quantitative study** show high scores in the average observance “by the employees themselves” (over 4 in a scale of 5). Moreover, participants believe that their observance of these values is higher than that of their colleagues and that the corporate observance is even lower. These results do not vary when we compare them by gender, age groups and professional discipline. This can be interpreted either as an overestimation of their own observance or a real interest, adherence and awareness of ethical values in these individuals. Some studies show differences between genders in the self-assessment of training on ethical matters and differences between self-evaluation and the evaluation of others in relation to clinical variables. We have not found similar studies to compare these results, but a study conducted by Donna Bobek and colleagues analyses [27] the differences between leaders and non-leaders in the evaluation of the ethical climate of organizations. They found that leaders tend to perceive that the ethical climate of the organization is significantly better than the non-leaders. However, when the non-leaders believe that they have a significant role in the upkeep of the ethical climate, these differences diminish [27, 28].

In our study we found no differences in compliance with the CE values according professional disciplines. This could be explained by considering that the CE defines the style of the CIH and a common ethical framework for all workers, but does not include the specificities of the professional disciplines. Making a comparison with an orchestra, the ethical code of the organization define the type of orchestra and the sheet music that all must share, but each member of the orchestra has to take into account the particularities of its own instrument.

If we analyse the nursing discipline, it should be noted the close relationship between the ethical sense of care and the principles of bioethics [29]. Perhaps for this reason, several studies have focused on the perspectives of nurses on their ethical codes: value, development, dissemination and implementation in daily practice [30–35]. Most of this work emphasizes the need for providing continuing ethics education, design ethical programs and evaluate the competence and ethical skills. Moreover, to make changes in the development of such codes, and the support at institutional level, since in many contexts appear to be ‘paper tigers’ with little or no impact in daily practice.

The **comments** on the values mirror the ethical climate of the organization, show several ethical dilemmas, suggestions to tackle the ethical challenges faced by the workers and the organization and express how they want to be and to work. Effective communication of the values of the CE, debate between workers and management, being clear about what compromises and attitudes are expected, they all contribute to decrease variability, uncertainties and conflicts and enhance satisfaction, as suggested by several authors [4, 36, 37]. Moreover, if the code is introduced in the culture of the organization and is assumed by the management it has more probabilities to succeed [20].

The involvement of employees of various levels of care and from different geographical areas supports the importance of ethics and the need to complete the CIH CE project. It also conveys the workers’ understanding of the organisation and their willingness to contribute their views on the ethical values of the CIH, which suggests a sense of compromise and of belonging to the organization [3].

The eight values that we finally included in the CIH CE originate from the CEVF (identified in the previous phase, also participative) after the analysis of the CEVF and the meetings of the Code of Ethics Commission and the CIH management. These values agree with the principles of bioethics and are clustered accordingly: autonomy (cooperation and integrity), justice (equity and responsibility), non-maleficence (respect and innovation) and beneficence (competence and trust) [38]. A study published in 2008 [20] compared the values chosen in health organisations from Europe, America and Asia and found that Europeans highlighted environmental issues and justice whereas for the Americans honesty was paramount [39], in agreement with the values of their respective health systems.

The CIH CE aims to guide in challenging situations and includes attitudes that should be encouraged. However, it also points at behaviours to be avoided since some of them are not easily identified by employees.

### Discussion on the values of the CIH Code of Ethics

**Competence** demands to keep up with the required knowledge, abilities and attitudes and a determination toward improvement. Competence is more than the sum of abilities. It also consists of cognitive, integrative, relational and affective functions [40] and more specifically, professionalism [41]. Few comments have been posted in the forum on this value, which obtained an average score of 4.3 in the questionnaire. However, there were comments on competence amidst comments on other values. It is possible that the lack of comments responds to competence being considered an essential value, something inherent to a health organization. In addition, in previous phases of preparation of the CE the value was “quality”, the illustrative case was somewhat difficult to understand and it was also the penultimate value.

**Confidentiality and respect** could be considered cross-values since they are integrated within other values. Confidentiality and respect are found in most corporate CE [42], in agreement with the enforcement of personal data protection in Europe [43].Indeed, trust, confidentiality and respect are intrinsic values of human competence that sustain all professional practice [2]. Confidentiality generates the trust and sense of security essential in healthcare [44]. Trust is also crucial toward employees’ perception of equity and justice of the organization [45]. Confidentiality obtained the highest score in the questionnaire and elicited the highest number of comments in the forum.

The crisis of values and lack of respect in human interaction are a current topic of discussion. **Respect** is the genuine interest to understand the ideas of others and to hold people as entities that need to be cared for. It also involves the acceptance of disagreement, working to the best of your ability and assuming a compromise of appreciation with the public, colleagues and the organization. This respect is based on three axes: respect for the freedom and dignity of patients; respect for the preferences and decisions of patients; and respect for the job and the decisions of professionals [46]. These three axes correspond to the categories of analysis identified in the comments about this value.

Kaptein’s review shows that 67 % CE include the value **responsibility** of organizations in relation to the quality of their products and services [39, 47]. Responsibility is a value present in all phases of production of the CIH CE, is part of the strategic plan and includes transparency. Responsibility implies good practice in healthcare, research, teaching and management. It also requires involvement with the vision and objectives of the organization.

**Teamwork** is essential in the health services. It requires cooperation, participation, critical thinking and personal involvement. With cooperation, complementarity and coordination of work employees develop their individual work capacity and learn to accept criticism, self-criticism, to respect the opinions of others, to listen and to be flexible. Teamwork is an opportunity for mutual learning, it facilitates the identification of employees and the organization, boosts the quality of work and contributes to the achievement of the objectives of professionals and the organization [48–50]. The participants of our study show a positive attitude toward teamwork. However, it is the value with the lowest score of the whole questionnaire. In addition, respondents highlight many hurdles in themselves and within the organisation to fully develop teamwork. These hurdles could be de basis of suggestions for improvement.

**Integrity** is an essential professional value. It is the compromise to act with honesty and coherence with professional and corporate values. It means to state clearly any conflict of interest and resolve them prioritizing service to the public [51]. Even though participants specify other issues on integrity, there is concern in this study about complicity with the pharmaceutical industry. Also, the workers highlight that in some cases the priority leans more toward achievement of objectives and incentives than on patient care.

**Equity** is a value included in the CE of all type of organizations in many countries [52]. Equity refers to fair treatment, considering the circumstances and set of values of every person. Equity relates to the relationship with workers, providers and stakeholders. Professional practice involves thinking about the equity and efficiency of each decision [51]. Participants believe that this value should improve in all areas of the organization. The CE should facilitate the standardization of practices and improvements in equity.

The entries in the forum, the input of the Code of Ethics Commission, a review of the literature and the opinion of management were all taken into account for the selection of values of the CE. The value **innovation** was included even though it did not appear in the comments since it is considered crucial to determine the essence of the organization.

#### Implications of findings

The results of the study have been crucial for the development of the current CE of the CIH and have added a positive spin, which according to some authors should facilitate its implementation [53]. The CE was approved on July 13, 2010 (http://www.gencat.cat/ics/infocorp/flpb/codietic/). The forum was open until 2013 as a meeting point and space for debate and suggestions on ethical values, with the aim to continue encouraging employee participation, an environment of shared ethical values, internal cohesion and relationships based on mutual trust. The employees’ contributions are crucial for the improvement, review, update and implementation of this CE, a responsibility which all stakeholders should share. Some authors and organizations [43, 54, 55] observe that the CE is a live tool that implies revision, reflection and change to adapt to new dilemmas, emerging challenges, to the progress of science and to social and political changes and thus increase the trust of the public [26].

The values of the CIH CE are a hallmark of the organization and professionals and enhance quality of care and professional satisfaction. They guide good clinical practice and humanity in relationships. It is essential to implement them individually and to share them for an effective teamwork so as to achieve improved relationships with other professionals and with patients. A practice consistent with these values obtains social approval and increases trust in the organization. While there is no doubt that the professional values existed already, the CIH CE presents them as a common goal since they have been chosen by the employees themselves, and it defines them explicitly. Thus, when an employee is faced with an ethical dilemma he/she can decide in accordance to the character, ethos and corporate identity, and does not need to rely solely on his/her individual opinion. A common framework of values also enhances the coherence of corporate decisions, public engagement and institutional policies.

#### Limitations of the study:

The number of entries posted in the forum for the questionnaire and comments is significant and show the point of view of workers of various professional disciplines, posts, levels of healthcare and geographical areas. However, in accordance with other studies [56] participation rate was low (approximately 10 % of employees responded). Moreover, participants were probably those employees more concerned about ethics. . However, the responses by those most concerned about these issues has made possible the identification of ethical challenges and has contributed suggestions for improvement. Here we should highlight the criticism toward management. Indeed, healthcare practitioners feel little appreciated by management, management does not react when faced with inappropriate attitudes and behaviours and they do not focus on the people that is in the frontline of healthcare, those in charge of patients which are, after all, the true essence of the CIH. In agreement with previous literature, participants consider that the management must adhere and set example in relation to the observance and dissemination of the CE.

Another limitation that we share with other studies [56] is the use of an ad-hoc questionnaire, since there is no standard to compare its psychometric properties. On the other hand, the accurate implementation of the pilot study supports the applicability and understandability of the questionnaire.

#### Future research

Further research should involve longitudinal studies with randomized sampling, the analysis of knowledge of workers about the CE and its applicability and use, by means of validated questionnaires and to observe the consistency of behaviour in relation to the CE. Results would be further supported by the inclusion of the opinions of the public and professional associations on the CE. Up-to-date systematic reviews and metasynthesis should be carried out. José Felix Lozano explains that the CE needs constant updates and a Corporate Ethics Commission to guarantee its effective implementation [37, 57].

This study shows that the CIH CE has been prepared with a broad participation and supported by management for the resolution of ethical dilemmas.

## Abbreviations

CE: code of ethics
CIH: Catalan Institute of Health
CEVF: code of ethics virtual forum
